# Identifying the Causes of Drivers’ Hazardous States Using Driver Characteristics, Vehicle Kinematics, and Physiological Measurements

**DOI:** 10.3389/fnins.2018.00568

**Published:** 2018-08-14

**Authors:** Ali Darzi, Sherif M. Gaweesh, Mohamed M. Ahmed, Domen Novak

**Affiliations:** ^1^Department of Electrical and Computer Engineering, University of Wyoming, Laramie, WY, United States; ^2^Department of Civil and Architectural Engineering, University of Wyoming, Laramie, WY, United States

**Keywords:** hazardous driver state, driving performance, physiological measurements, human factors, affective computing

## Abstract

Drivers’ hazardous physical and mental states (e.g., distraction, fatigue, stress, and high workload) have a major effect on driving performance and strongly contribute to 25–50% of all traffic accidents. They are caused by numerous factors, such as cell phone use or lack of sleep. However, while significant research has been done on detecting hazardous states, most studies have not tried to identify the causes of the hazardous states. Such information would be very useful, as it would allow intelligent vehicles to better respond to a detected hazardous state. Thus, this study examined whether the cause of a driver’s hazardous state can be automatically identified using a combination of driver characteristics, vehicle kinematics, and physiological measures. Twenty-one healthy participants took part in four 45-min sessions of simulated driving, of which they were mildly sleep-deprived for two sessions. Within each session, there were eight different scenarios with different weather (sunny or snowy), traffic density and cell phone usage (with or without cell phone). During each scenario, four physiological (respiration, electrocardiogram, skin conductance, and body temperature) and eight vehicle kinematics measures were monitored. Additionally, three self-reported driver characteristics were obtained: personality, stress level, and mood. Three feature sets were formed based on driver characteristics, vehicle kinematics, and physiological signals. All possible combinations of the three feature sets were used to classify sleep deprivation (drowsy vs. alert), traffic density (low vs. high), cell phone use, and weather conditions (foggy/snowy vs. sunny) with highest accuracies of 98.8%, 91.4%, 82.3%, and 71.5%, respectively. Vehicle kinematics were most useful for classification of weather and traffic density while physiology and driver characteristics were useful for classification of sleep deprivation and cell phone use. Furthermore, a second classification scheme was tested that also incorporates information about whether or not other causes of hazardous states are present, though this did not result in higher classification accuracy. In the future, these classifiers could be used to identify both the presence and cause of a driver’s hazardous state, which could serve as the basis for more intelligent intervention systems.

## Introduction

Many traffic accidents are caused, at least partially, by the driver being in a hazardous mental or physical state. Road accidents caused by fatigue, for example, resulted in an estimated 800 deaths and 41,000 injuries in the United States in 2015 (The National Highway Traffic Safety Administration [NHTSA], 2015). As another example, traffic accidents caused by distracted driving resulted in 1.25 million deaths worldwide in 2015, with an estimated 3,477 deaths and 391,000 injuries in the United States alone (National Highway Traffic Safety Administration [NHTSA], 2016).

One way to reduce the number of deaths and injuries caused by hazardous driver states (HDS) would be via automated systems that monitor drivers’ mental and physical state and intervene in dangerous situations. Several such systems have been proposed (Gallahan et al., 2013; Zhang et al., 2017), but have not yet attained sufficient accuracy for widespread implementation. An important issue with such automated intervention systems (as described in more detail later in this section) is that they generally do not identify the cause of the hazardous state (e.g., drowsiness vs. stress) and therefore cannot tailor their response to the specific issue at hand. Thus, the goal of this study is to develop an automated system that not only detects HDS, but can also identify specific causes of hazardous states (e.g., sleep deprivation, cell phone use) based on driver characteristics, vehicle kinematics, and driver physiology.

### Causes of Hazardous Driver States

Hazardous driver states can be related to either physical/physiological (e.g., fatigue) or cognitive/affective (e.g., anger) conditions. These conditions can be intrinsic (e.g., sleep deprivation) or extrinsic (e.g., adverse weather). However, most causes of HDS have both physical and mental components and are related to both intrinsic and extrinsic processes; furthermore, the contribution of different factors may be further affected by driver characteristics such as driving experience or personality. We thus present a few common causes of HDS, which will also be the focus of our study, without delving deeply into the underlying processes.

#### Distractions

Perhaps the most infamous HDS are distractions caused by secondary tasks performed in addition to driving. Distractions can lead to catastrophic situations since drivers often need up to 7–12 s to regain situational awareness (Lu et al., 2017). For example, cell phone use is recognized as a major problem in driving (Collet et al., 2010; Yang and Parry, 2014; Haque and Washington, 2015), and results in both visual distractions (no longer watching the road) (Recarte and Nunes, 2003) and cognitive distractions (no longer thinking about driving) (Hwang et al., 2014). Similarly, other in-vehicle technologies such as radio or navigation systems are major potential distraction sources (Beede and Kass, 2006; Horrey et al., 2017). Thus, many studies have focused on the ergonomic design of in-vehicle technologies (François et al., 2017) and the development of novel technologies to reduce distraction [e.g., auditory menu navigation (Jeon et al., 2015)]. Distractions influence the driver’s physiology (Hirayama et al., 2016) and vehicle kinematics (Liang and Lee, 2010) and significantly increase the risk of crash or near-crash among all drivers, particularly novices (Klauer et al., 2014).

#### Fatigue

Any driving situation becomes more difficult to handle if the driver is fatigued or sleep-deprived, as fatigue affects both driver physiology (Zhang et al., 2014; Chuang et al., 2018) and vehicle kinematics (Guo et al., 2016). It has been shown to have a larger effect on inexperienced and elderly drivers than experienced and young ones (Li et al., 2016). Sleep deprivation is a special case of fatigue and involves cognitive impairment or lower efficiency due to lack of sleep (Yang et al., 2009). Proper design of vehicle seats is effective in reducing the fatigue generated by prolonged static posture (Sales et al., 2017), and actions such as dynamic movement of the backrest angle can also reduce fatigue (Rhimi, 2017).

#### Demanding Driving Conditions

Even if a driver is rested and fully focused on driving, difficult driving conditions such as blizzards introduce a high level of mental demand. During such conditions, drivers may devote all their mental resources and still not be able to drive effectively (Recarte and Nunes, 2003; Shakouri et al., 2018). High workload level can also lead to stress, which affects the driver’s behavior and physiology (Healey and Picard, 2005; Stuiver and Mulder, 2014). For example, eye movements are faster and heart rate (HR) is higher in stressful conditions (Healey and Picard, 2005). Even if the driver is not stressed, the increased workload can increase the probability of other HDS such as distraction (Hwang et al., 2014; Kandemir et al., 2016). Thus, many studies have used workload as an indirect indicator of driver impairment (Čegovnik et al., 2018; Rusnock and Borghetti, 2018).

#### Mood, Personality, and Other Intrinsic Factors

Finally, the probability of HDS depends on the driver’s general mood as well as intrinsic factors such as personality. For example, negative moods increase the frequency of risky behaviors as well as drivers’ perception of risk (Hu et al., 2013). As another example, distracted driving is more common in conscientious teens and extraverted older adults (Parr et al., 2016). Furthermore, older drivers may find it more difficult to complete certain tasks due to longer reaction time (Pierce and Andersen, 2014), but are also likely to have more experience that makes driving easier in general (Williams, 2003).

### Assessment of Hazardous Driver States

Regardless of their causes, HDS can be assessed using three methods: self-reported driver characteristics, physiological measurements, or vehicle kinematics measurements.

#### Driver Characteristics

Driver characteristics are frequently used to report psychological states. For instance, the State Affect Questionnaire (STAQ) (Larsen and Diener, 1992), Perceived Stress Scale (PSS-10) (Cohen et al., 1983), and NASA Task Load Index (NASA-TLX) (Hart and Staveland, 1988) are used to assess mood, stress, and workload, respectively. However, they generally cannot be used during driving, as they would serve as a distraction.

#### Physiological Measures

Physiological measures can unobtrusively quantify psychological states by measuring the physiological responses to such states. They include the electrocardiogram (ECG) (Jung et al., 2014), which records HR (Collet et al., 2009), galvanic skin response (GSR), which records the activity level of the skin’s sweat glands (Bongiorno et al., 2017; Rodriguez-Guerrero et al., 2017), respiration rate (RR) (Healey and Picard, 2005), skin temperature (ST) (Kajiwara, 2014), eye gaze (Fletcher and Zelinsky, 2009), blink frequency (He et al., 2017), electroencephalography (EEG) (Mühl et al., 2014; Mu et al., 2017), and others. They are quantitative and can be recorded in a real-time manner without the user’s active involvement, but are often affected by noise and difficult to interpret (Healey and Picard, 2005).

#### Vehicle Kinematics Measurements

Vehicle kinematics measurements include measures such as reaction time (Guo et al., 2016; Choudhary and Velaga, 2017), the force applied to the gas pedal, longitudinal speed (Jun et al., 2011), rotation of the steering wheel (Zheng and Hansen, 2017), and the lateral lane position (distance from center of lane) (Beede and Kass, 2006; Sun et al., 2015). All of these can be used to detect or predict different HDS [e.g., distraction (Liang and Lee, 2010)].

### Automated Classification of Hazardous Driver States

The major scientific challenge in HDS analysis is how to convert multiple potentially unreliable measurements into an estimate of the type of HDS and/or the cause of a hazardous state. This can be achieved using automated classification algorithms. The most common basis for such automated classification algorithms have been measures of vehicle kinematics such as steering wheel rotation (Zheng and Hansen, 2017) and global positioning system data (Singh et al., 2016), which are used to detect distraction and lane changes, respectively. Perhaps the first major study in using physiological signals for driver monitoring was done by Healey and Picard (2005), who used ECG, RR, GSR, and EEG to classify stress levels. Since then, a number of studies have used physiological signals to detect workload levels (Fan et al., 2017) or distracted driving (Hirayama et al., 2016; Liu et al., 2016). A small number of studies have also taken driver characteristics into account (Guo and Fang, 2013; Rahemi et al., 2017). While these studies have achieved promising results, it has been shown that the combination of vehicle kinematics, physiological measurements, and driver characteristics is the most effective way to detect HDS such as inattention (Dong et al., 2011) and drowsiness (Sahayadhas et al., 2012).

Although many studies were able to detect the presence of HDS, most of them only used a single way of inducing a hazardous state – e.g., cell phone use (Beede and Kass, 2006), high speeds (Kidd and Buonarosa, 2017) or driving in dense traffic (Fastenmeier and Gstalter, 2007). While a few studies do use multiple ways to induce hazardous states in drivers (e.g., Liang and Lee, 2010), they do not attempt to identify the cause of the hazardous state. Furthermore, many real-world and simulated driving studies that involve physiological measurements focus on a single session (Healey and Picard, 2005; Kidd and Buonarosa, 2017). This may not capture within-subject variability (e.g., the same driver may have different reactions at different times of day), so studies should ideally involve multiple sessions (as done, e.g., with three drivers by Healey and Picard, 2005).

### Study Contribution

The contribution of the present study is as follows: Unlike most previous studies, we exposed drivers to four different causes of HDS (mild sleep deprivation, adverse weather, cell phone use, and high traffic density), and collected three different types of information: vehicle kinematics, physiological measurements (RR, ST, GSR, and ECG), and driver characteristics (personality, mood, and stress level). We then created different classifiers to automatically identify the presence or absence of each of these four causes of HDS based on different types of information. The research questions were:

– **RQ1**: Can the obtained features be used to automatically classify different specific causes of HDS? Previous studies have mainly focused on a single way of inducing a hazardous state, but this is not necessarily optimal; for example, intelligent cars should intervene differently if the driver is sleep-deprived than if the driver is texting on their smartphone, necessitating the classification of specific causes of HDS. We **hypothesized** that some causes of HDS will be easier to classify than others, with the highest classification accuracy achieved for sleep deprivation.
– **RQ2**: Are certain types of information better at classifying certain causes of HDS, and can different types of information (kinematics, physiology, and driver characteristics) be combined for more accurate classification? Many recent studies (e.g., Mu et al., 2017; Zheng and Hansen, 2017) focus on a single measurement (e.g., EEG or vehicle kinematics), but this is not optimal. For example, even if, e.g., respiration can be used to detect a specific HDS, it is possible that the same HDS could be identified more easily and cheaply by, e.g., measuring vehicle drift using sensors that are already built into the car and would not inconvenience the driver. As another example, while physiology or vehicle kinematics may be inaccurate when detecting a specific HDS on their own, combining both types of information may lead to much more accurate classification. We **hypothesized** that, for all four causes of HDS, combining the three different types of information will lead to higher classification accuracy than using only a single type of information.
– **RQ3**: When multiple causes of HDS may be present, does providing information about some causes allow more accurate classification of other causes? For example, if trying to detect the use of a cell phone, will a classifier be more accurate if it already knows whether the driver is sleep-deprived and/or driving in poor weather conditions? This would pave the way for, e.g., two-stage classification where an intelligent system first identifies the driving environment and only then classifies the driver’s mental state. We **hypothesized** that, for classifying each cause of HDS, the accuracy will be higher if information about the other three causes of HDS is provided as an additional input.

These research questions were investigated over multiple sessions, going beyond the single-session studies that are common in this field (e.g., Healey and Picard, 2005; Zheng and Hansen, 2017).

## Materials and Methods

This section is divided into seven subsections that describe the hardware and study setup, the study protocol, the driver’s characteristics, the vehicle kinematics measures, the physiological measures, the classification methods, and the statistical validation methods.

### Study Setup

**FIGURE 1:**
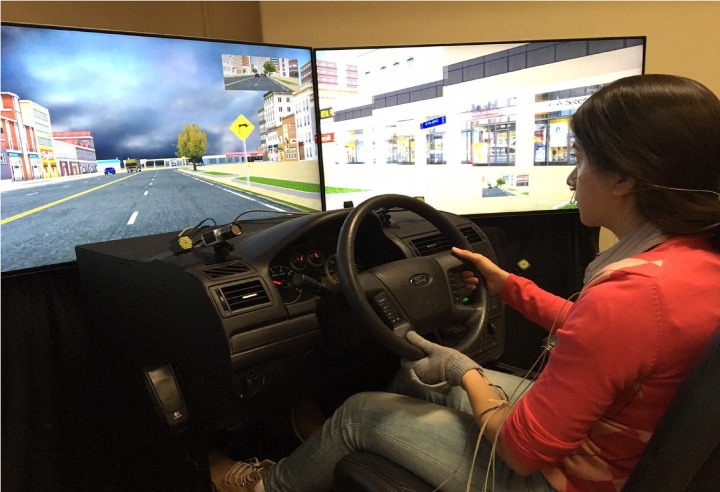
The study setup: WYOSIM simulator and physiological sensors. Written informed consent from the subject has been obtained for publication of the photograph.

The simulated driving scenarios were implemented in the University of Wyoming driving simulator lab (WYOSIM). WYOSIM is based on an open-cab 2004 Ford Fusion simulator developed by Realtime Technologies Inc (**Figure 1**). The simulator is mounted on a three-degree-of-freedom D-Box motion base (roll, pitch, and heave) with four electromechanical linear actuators. Additionally, three 55-inch high-definition screens provide a 150-degree forward and side field of view.

**FIGURE 2:**
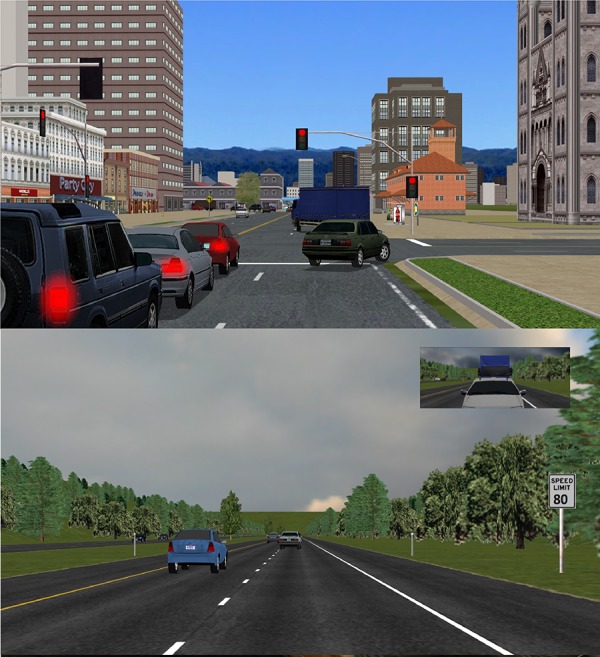
Town scenario **(Top)** and highway scenario **(Bottom)**.

Two different driving environments were simulated for the study: a city and a rural highway (**Figure 2**). The city includes multiple junctions with traffic lights as well as denser traffic than the highway. Furthermore, the highway has a posted speed limit of 80 miles per hour (mph) while the city has a posted speed limit of 35 mph.

Since environmental light levels can have a significant effect on the driver, both the city and highway scenarios can be set either in daylight or at night. This was done by setting the simulated time to 10 pm for night sessions and 10 am for day sessions, resulting in a darker environment and activated headlights in night sessions. Furthermore, as driving performance is affected by weather (e.g., snowy weather reduces visibility and causes slippery roads), the scenarios can have different weather conditions: clear and sunny or foggy and snowy. In snow, visibility is significantly reduced, and friction between the tire and road is reduced to 60% of the clear-weather value. The friction level in snowy weather was selected empirically in pilot tests.

### The Protocol of the Study

The study was approved by the University of Wyoming Institutional Review Board (protocol #2016062201232). Twenty-five people were recruited at the beginning of the study. Four chose to discontinue participation after the first session, resulting in 21 valid healthy participants (25.1 ± 8.7 years old, six females). All subjects gave written informed consent in accordance with the Declaration of Helsinki. They were enrolled in a four-session study protocol. Of the four sessions, two were meant to mimic drowsy (mildly sleep-deprived) driving and were held in the early morning while it was still dark outside, with the participant instructed to have less than 6 h of sleep the preceding night. In these sessions, only night scenarios were used in WYOSIM. The other two sessions were meant to mimic alert driving and were held between 10 am and 5 pm, with the participant instructed to have at least 7 h of sleep the preceding night. In these sessions, only day scenarios were used in the WYOSIM. The alert (day) and drowsy (night) sessions were held in random order.

**FIGURE 3:**
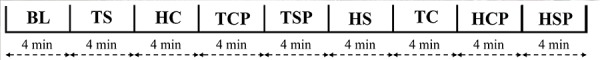
An example session protocol. BL, baseline; T, town; H, highway; C, clear weather; S, snowy weather; P, using cell phone; without P, no cell phone. The eight scenarios after the baseline were presented in random order, and the presented example is only one of the possible orders.

The first session began with a 10-min practice scenario where participants familiarized themselves with the driving simulator. Then, participants were asked to relax while physiological measures were taken for a 4-min ‘baseline’ period. This baseline period was followed by eight scenarios that represent all possible combinations of traffic density (high/low), weather (sunny/snowy), and cell phone use (phone/no phone), in random order. In this paper, adverse weather and dense traffic are considered to be ‘environmental’ causes of HDS. In the ‘cell phone’ scenarios, participants were asked to use their phone to send messages, browse websites, or watch videos (but not make phone calls). The cell phone was considered the final, fourth cause of HDS. Each scenario was presented for 4 min, and participants were asked not to talk during the scenarios. A brief break (2 min) was given after each scenario for two purposes: to fill out the NASA-TLX questionnaire (se section “Driver Characteristics”) and to allow the effect of the scenario to be “washed out.” Participants were allowed to take a longer break in case of dizziness, headache or discomfort. An example session protocol is shown in **Figure 3**, though readers should note that this was only one possible order of scenario presentation and that scenarios were presented in random order within a session.

For sessions 2–4, the study protocol was identical to the one in the first session, except without the practice scenario. The protocol thus includes four different causes of HDS: sleep deprivation, poor weather, high traffic density, and the use of cellphones. Sleep deprivation was present in two entire sessions and absent in the other two sessions; each of the other causes of HDS was present in half the scenarios within each session and absent in the other half of the scenarios. Each participant therefore experienced 32 different scenarios (eight per session), with each scenario including between zero and four causes of HDS.

### Driver Characteristics

Five types of driver characteristics were collected using questionnaires:

(1) The participant’s basic information was collected using an initial questionnaire that asks about age, gender, dominant hand, driving experience (in years), height, and weight.
(2) Personality was assessed with the International Personality Item Pool (IPIP) (Goldberg et al., 2006), which covers five traits: extraversion, neuroticism, agreeableness, openness, and conscientiousness. It was filled out at the start of the first session, as the result is not expected to change over time.
(3) Stress level over the last month was assessed using the PSS-10 (Cohen et al., 1983) at the start of the first session. Since all sessions took place within approximately a week, the result was again not expected to change from session to session.
(4) The participant’s mood over the last 24 h was assessed using the STAQ (Larsen and Diener, 1992), which covers four psychological aspects: negative affect, positive affect, activated, and inactivated. It was filled out at the start of each session.
(5) Workload was assessed using the NASA-TLX (Hart and Staveland, 1988), which covers six workload aspects: mental demand, physical demand, temporal demand, performance, effort, and frustration. It was applied after each driving scenario.

The NASA-TLX was used as a validation measure to check whether the different scenarios were able to induce different workload levels. Data from the other questionnaires (basic information, personality, stress, and mood) are collectively referred to as ‘driver characteristics’ and were used as inputs to classify the presence or absence of different causes of HDS. Since they were collected only once per participant or once per session, we did not expect them to be able to differentiate between different scenarios on their own; however, they may be able to enhance classification of vehicle kinematics or physiological measures by providing a way to individualize the classifier to a particular participant or session.

### Vehicle Kinematics

The WYOSIM records numerous signals related to vehicle kinematics with a frequency of 60 Hz. We selected eight signals that contain information about vehicle kinematics but do not reveal any information about the simulated environment (e.g., weather). **Table 1** presents these signals. From the raw signals, we extracted multiple features that were calculated over each 4-min scenario interval. For each raw measure, we calculated its mean, standard deviation, and the mean absolute value of the first derivative (also referred to as ‘fluctuation’). This resulted in a total of 24 vehicle kinematics features for each scenario.

**Table 1:** Selected driving behavior signals.

	Measure	Definition
1	Throttle force	Force applied to gas pedal
2	Lane number	Right or left (binary)
3	Lateral lane position	Distance between middle line of the car and middle line of the lane
4	Road offset	Distance between middle line of the car and middle line of the road
5	Longitudinal velocity	Velocity in forward direction
6	Vertical velocity	Up-down velocity
7	Slip level of front tires	Level of slip in range of 0–1
8	Slip level of rear tires	Level of slip in range of 0–1


### Physiological Measures

**FIGURE 4:**
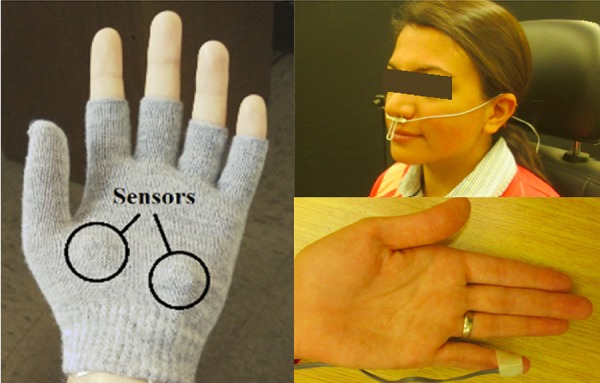
Placement of GSR sensors on the palm of the hand **(Left)**. Respiration sensor placement below the nose **(Top Right)**. Skin temperature sensor is taped to the distal phalanx of the little finger **(Bottom Right)**. Written informed consent from the subject has been obtained for publication of the photograph.

Four physiological signals were recorded from the participants using a g.USBamp signal amplifier and associated sensors (g.tec Medical Engineering GmbH, Austria), as shown in **Figure 4**. ECG was recorded using four electrodes on the body (two on the chest, one on the shoulder, and one on the abdomen) as recommended by the manufacturer of the g.USBamp. Respiration was recorded using a thermistor-based sensor in front of the nose and mouth. ST was recorded using a small sensor attached to the distal phalanx of the little finger of the non-dominant hand using tape. GSR was recorded via two electrodes (g.GSRsensor2, g.tec) attached to the palm of the non-dominant hand using a fingerless glove. While GSR electrodes are usually placed on the index and middle fingers, this would prevent participants from driving, and the palm approach was used instead based on recommendations from the literature (Boucsein, 2012). The two electrodes monitor skin conductance, which rapidly increases and smoothly decreases due to activity of the sweat glands.

The sampling frequency for all signals was 512 Hz. For RR, GSR, and ST, a band-pass filter (0–30 Hz) was used to reduce high-frequency noise. For ECG, a high-pass filter (cutoff at 5 Hz) was used to eliminate low-frequency noise, and a 60-Hz notch filter was used to remove electrical interference.

For each 4-min scenario, a total of 17 features were extracted from the four physiological signals as follows:

**RR:** The mean RR (number of complete breathing cycles per minute), the standard deviation of RR, and the root-mean-square of successive differences of respiration periods were calculated.

**ST:** Mean ST and the difference in ST between the first and last second of the scenario were calculated.

**GSR:** The GSR can be divided into two components: the tonic (low-frequency) and phasic (high-frequency) component. For the tonic component, the mean GSR and the difference in GSR between the first and last second of the scenario were calculated. The phasic component consists of discrete skin conductance responses, and we calculated the number of responses, the mean response amplitude, and the standard deviation of response amplitude (Boucsein, 2012).

**ECG:** Four time-domain features were calculated: mean HR, standard deviation of inter-beat intervals, and the median and absolute value of the gradient of the ECG signal. Furthermore, three frequency-domain features were calculated: power of low frequencies (LF), power of high frequencies (HF) and the power ratio of low to high frequencies (LF/HF). The LF range is 0.04–0.15 Hz while the HF range is 0.15–0.4 Hz (Task Force of the European Society of Cardiology and the North American Society of Pacing, and Electrophysiology, 1996).

### Classification and Validation

Two types of classification were performed in the study: simultaneous classification of all four causes of HDS and classification of each cause of HDS given information about the other three. In simultaneous classification, separate classifiers were used to classify the presence vs. absence of each cause of HDS without any information about the other three (which could also be present or absent in any given 4-min scenario). In the other classification scheme, separate classifiers were also used to classify the presence vs. absence of each cause of HDS; however, the classifier for each cause of HDS was provided with three additional binary features that indicated the presence (1) or absence (0) of each of the other three causes of HDS.

Aside from this difference, both types of classification were performed using the same normalization, feature selection, classification and validation methods.

#### Normalization

As the measured features may vary between participants and sessions (e.g., some participants have a higher baseline HR than others), they should be normalized to ensure a similar range for all measurements. This is a standard step in automated classification of physiological measurements, and has a major effect on classification accuracy (Novak et al., 2012). In this study, the physiological and vehicle kinematics features were normalized for each session. For physiological features, the obtained baseline value of a feature in each session was first subtracted from each scenario value of that feature for that session. Then, the maximum and minimum of each feature during a session were used to normalize the feature by subtracting the minimum value and dividing the result by the difference between the maximum and minimum values. The same procedure was used for vehicle kinematics features, but without subtracting the baseline value (which does not exist for vehicle kinematics).

#### Feature Selection

Since over 60 features were extracted from the different data sources, using all of them in classification would likely lead to overfitting, and only the most relevant ones should be selected (Novak et al., 2012). Prior to training a classifier on a particular dataset, we thus used stepwise forward feature selection with a threshold significance level of 0.05 (Webb and Copsey, 2011) to reduce the dimensionality of the data. In cases where no feature met the 0.05 criterion for inclusion (which occurred only for vehicle kinematics features and alert-drowsy classification), the threshold was raised to 0.1.

#### Classification

In total, the three different data sets provide us with 61 possible input features (17 physiological, 20 driver characteristics, and 24 vehicle kinematics) for classification. The classification inputs must be converted into four binary outputs that represent the presence or absence of each source of HDS. A total of 672 samples (21 participants × 4 sessions × 8 scenarios) are available to train the classifiers.

Three classifiers were tested: support vector machines (SVMs) (Press et al., 2007), logistic regression (LR) (Hosmer et al., 2013), and decision trees (DTs) (Kamiński et al., 2017).

Support vector machine maps labeled training data to higher dimensions and uses a hyperplane to classify them. The mapping method is defined by a kernel (Press et al., 2007), and we tested three different kernels: linear, quadratic and medium Gaussian. Whenever SVM results are presented in this paper, the kernel used is also given.

Logistic regression uses labeled training data to create a LR model with a possible output range of 0–1. An input data point is then classified as one class for model outputs below 0.5 and as the other class for model outputs above 0.5 (Hosmer et al., 2013).

Decision tree is a flowchart-like structure with several nodes and branches. Each node performs a threshold test on a single feature, and the two resulting branches indicate whether the feature was above or below the threshold. If a node has no branches connected to it, it is called an end node. Based on the number of nodes and branches, a tree can be simple, medium or complex (Kamiński et al., 2017). In this study, DT with different complexity levels were used for classification.

#### Validation

The method of leave-one-out cross-validation was used to validate the designed classifiers (Webb and Copsey, 2011). In cross-validation, the data is partitioned into *k* subsets. Classifiers are trained using data from *k -* 1 subsets, then validated on the remaining subset. The validation is repeated *k* times, with each subset acting as the validation subset once. The mean accuracy for classification over all *k* subsets is reported as the final result.

As a secondary result of the validation, the significance level of each selected feature is given. The significance levels are the result of an *F*-test that the stepwise algorithm uses to find the best features.

### Statistical Validation of Causes of HDS

Finally, repeated-measures analysis of variance (RM-ANOVA) was used to find the effect of the four causes of HDS on the six aspects of driver workload measured by the NASA-TLX. This was done by using the presence/absence of the four causes of HDS as within-subject factors, and serves as a validation that different workload levels were actually induced by the different scenarios.

## Results

We first analyzed the NASA-TLX results to verify that different workload levels were successfully induced with our driving environments, as described in Section “Effect of Causes of HDS on NASA-TLX Scores.” Section “Independent Classification of Each Cause of HDS” then uses all combinations of three features sets (driver characteristics, vehicle kinematics, and physiology) to independently classify the presence or absence of four causes of HDS (cell phone use, sleep deprivation, low vs. high traffic density, clear vs. snowy weather). It also presents the most relevant features for classification, as selected by stepwise feature selection. Section “Classification of Each Cause of HDS Given Information About the Other Three Causes” then presents the results of classifiers that already know the presence or absence of three causes of HDS and attempt to classify the presence or absence of the fourth cause of HDS given this information.

### Effect of Causes of HDS on NASA-TLX Scores

**Table 2** shows the significance levels for within-subject effects of cell phone use, sleep deprivation, traffic density, and weather on different aspects of the NASA-TLX. As **Table 2** demonstrates, using a cell phone while driving significantly affects all aspects of workload. Sleep deprivation, on the other hand, increases frustration and results in worse perceived performance.

**Table 2:** Significance levels for the effect of each cause of hazardous driver state on the different aspects of the NASA-TLX questionnaire.

	Cell phone	Alert vs. drowsy	Highway vs. town	Snowy vs. clear
Mental demand	**0.001**^∗^	0.269	0.344	**0.007**^∗^
Physical demand	**0.001**^∗^	0.667	0.606	**0.063**
Temporal demand	**0.001**^∗^	0.316	**0.099**	**0.078**
Performance	**0.001**^∗^	**0.080**	0.126	**0.064**
Effort	**0.001**^∗^	0.146	0.139	**0.068**
Frustration	**0.001**^∗^	**0.070**	0.682	0.170
Overall score	**0.000**^∗^	0.321	0.302	**0.018**


*Bolded values indicate *p* < 0.1 while asterisks indicate *p* < 0.01*.

### Independent Classification of Each Cause of HDS

**Table 3** presents the classification accuracies for independent classification of each cause of HDS using different combinations of input feature sets (driver characteristics, vehicle kinematics, and physiology). Driver characteristics can differentiate between alert and drowsy sessions since the mood questionnaire was filled out at the start of each session, but cannot identify other causes of HDS (which change within a single session, unlike driver characteristics). On the other hand, either physiology and vehicle kinematics can effectively discriminate between high and low traffic density (with accuracies over 80% for both feature sets), and vehicle kinematics can discriminate between snowy vs. clear weather (though only with an accuracy of 71.2%). The combination of all three feature sets results in the highest classification accuracies, though they are sometimes not much higher than using only a single feature set; for example, when classifying snowy vs. clear weather, the accuracy is 71.5% using all three feature types and 71.2% using vehicle kinematics alone.

**Table 3:** Independent classification of the four causes of hazardous driver states: accuracies for different combinations of features.

	Cell phone	Alert vs. drowsy	Highway vs. town	Snowy vs. clear
Physiology	81.8%	55.2%	86.8%	56.8%
Characteristics	–	98.8%	–	–
Vehicle kinematics	64.3%	53.1%	83.3%	71.2%
Physiology, characteristics	81.8%	98.8%	86.8%	56.5%
Physiology, vehicle kinematics	82.3%	55.2%	91.4%	71.5%
Characteristics, vehicle kinematics	64.6%	98.7%	83.3%	71.5%
All	82.3%	98.8%	91.4%	71.5%

**FIGURE 5:**
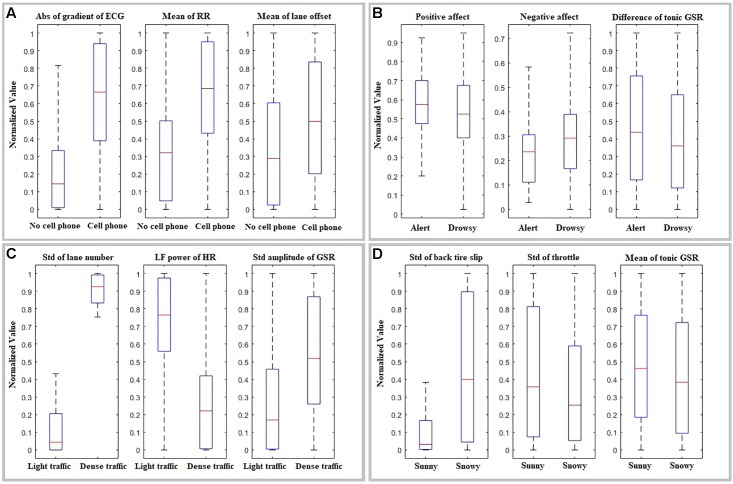
Box plots of the best selected features for independent classification of **(A)** cell phone vs. no cell phone, **(B)** drowsy vs. alert **(C)** low vs. high traffic density, **(D)** sunny vs. snowy weather. The baseline value of physiological data is subtracted and all data is normalized within a session by [data – minimum (session)]/[maximum (session) – minimum (session)]. Abs, absolute value; ECG, electrocardiogram; RR, respiration rate; LF, low-frequency; HR, heart rate; GSR, galvanic skin response; Std, standard deviation.

**Table 4** presents more detailed results of classification using all three feature sets. Specifically, it presents the best classifier and the best three features for each cause of HDS. The significance level of each feature, as calculated by stepwise selection, is also given. Furthermore, **Figure 5** presents box plots of each selected feature in the presence and absence of each cause of HDS (cell phone use, sleep deprivation, weather, and traffic density). Note that these box plots represent all scenarios when a specific cause of HDS was present, and the other causes may be either present or absent. More detailed examples of a few representative classifiers are provided in the **Supplementary Material**.

**Table 4:** Independent classification of the four causes of hazardous driver states: accuracies and best features when all three feature sets (physiology, vehicle kinematics, driver characteristics) are used as input features.

	Accuracy	Best classifier	Three best features	*P*-Value
Phone vs. no phone	**82.3%**	LR	Abs [gradient (ECG)]	*P* < 0.001
			Mean of respiration rate	*P* < 0.001
			Mean of lateral lane position	*P* < 0.001
Alert vs. drowsy	**98.8%**	Ensemble boosted DT	Negative affect	*P* < 0.001
			Positive affect	*P* < 0.001
			Difference of tonic GSR	*P* = 0.02
Low vs. high traffic density	**91.4%**	LR	Std lane number	*P* < 0.001
			Low-frequency power of heart rate	*P* < 0.001
			Std amplitude of GSR	*P* < 0.001
Snowy vs. clear	**71.5%**	SVM linear kernel	Std of rear tire slip	*P* < 0.001
			Std of throttle	*P* < 0.001
			Mean of tonic GSR	*P* = 0.018


*Abs, absolute value; ECG, electrocardiogram; Std, standard deviation; GSR, galvanic skin response*.

### Classification of Each Cause of HDS Given Information About the Other Three Causes

**Table 5** presents the classification accuracies for classification of each cause of HDS using different combinations of input feature sets (driver characteristics, vehicle kinematics, and physiology) as well as information about the presence or absence of the other three causes of HDS. Most accuracies are similar to those observed in the previous section where the presence/absence of the other three causes was not known (**Table 3**). Some accuracies are even slightly lower than in **Table 3**, which is likely due to the increased dimensionality of the problem – the three additional features (presence of other causes of HDS) are not informative enough to offset the increased number of features.

**Table 5:** Classification of each cause of hazardous driver state given information about the presence or absence of the other three causes: accuracies for different combinations of features.

	Cell phone	Alert vs. drowsy	Highway vs. town	Snowy vs. clear
Physiology	81.8%	55.3%	86.8%	56.8%
Characteristics	–	100%	–	–
Vehicle kinematics	64.8%	53.3%	83.3%	70.1%
Physiology, characteristics	81.8%	99.6%	86.8%	56.5%
Physiology, vehicle kinematics	82.8%	55.3%	91.3%	70.1%
Characteristics, vehicle kinematics	64.5%	100%	83.3%	70.2%
All	82.9%	100%	91.9%	70.8%


## Discussion

A positive overall result of our study is that the presence or absence of different causes of HDS can be classified with accuracies ranging from 70% (snowy vs. clear weather) to nearly 100% (sleep deprivation), regardless of whether other causes of HDS are present or not. However, as discussed in the next sections, not all feature types are equally informative, and the choice of classifier also affects accuracy.

### Identifying Different Causes of HDS

As seen in **Tables 3**, **4**, not all causes of HDS can be classified using the same features – some features are more effective at identifying certain causes.

#### Cell Phone Use

Cell phone use was most visible in the gradient of the ECG signal, the mean RR, and the mean lateral lane position. The effect on lateral lane position is not surprising, as using a cell phone makes it more difficult to focus on the road and should result in the driver not following the road as effectively (Papadakaki et al., 2016). The effect on RR, on the other hand, is an indicator of increased workload and may also be promising for automated identification of causes of HDS. Increased RR may allow HDS due to cell phone use to be detected before the driver begins dangerously drifting toward the edge of the lane. Finally, the gradient of ECG presents an interesting result: as this is the actual ECG signal (rather than the HR signal), these changes were likely caused by skeletal muscle artifacts created by arm gestures. While such artifacts are commonly filtered out in physiological data analysis, removing the ECG gradient decreased the accuracy from 82.3 to 74.3%. In this case, the artifacts thus serve a useful function in classification by providing information about body gestures without the need for camera monitoring.

#### Sleep Deprivation

The alert and drowsy sessions can easily be distinguished using driver characteristics – specifically, mood (negative and positive affect). The more negative mood in drowsy sessions likely also influenced physiological responses. This suggests that it would be worth including estimates of the driver’s overall mood as an input to our classifiers, which supports our hypothesis that combining different types of information will lead to higher classification accuracy. While it would likely not be practical to capture mood using questionnaires, it could potentially be done with physiological measurements – longer-term measurements (over periods of up to a few hours) have been used to identify overall mood and stress levels (Valenza et al., 2016) and could serve as a useful complement to the shorter-term measurements used in our study. Other than tonic GSR, physiological measurements, and vehicle kinematics did not make a useful contribution to classification.

#### Driving Environment

The standard deviation of the lane number is almost four times higher in dense traffic scenarios compared to light traffic, indicating that drivers changed their lane much more frequently. This does not necessarily indicate increased workload or decreased focus, and is likely only related to the need to, e.g., make left turns more often. However, features derived from HR and GSR likely indicate increased workload due to more cars on the road and other environmental features of a town like more intersections and more traffic lights.

#### Weather

Driving in snowy/foggy weather was primarily indicated by increased tire slip due to decreased friction, as well as by different participant behavior with regard to pushing the gas pedal. Weather was the most difficult to classify, likely because of large interpersonal differences in reactions: some participants slowed down in response to poor weather and had an unproblematic experience while others drove at the same speed as in sunny weather and consequently experienced high tire slip or even crashes.

Though different data types are more effective for different causes of HDS, we can nonetheless ask whether a single data type would be sufficient for multiple causes of HDS. Based on **Table 3**, it is our opinion that neither vehicle kinematics nor physiology are sufficient on their own: while vehicle kinematics can classify environmental causes of HDS (e.g., weather), physiology is more effective at identifying, e.g., cellphone use. Even driver characteristics (primarily mood, which could be estimated from physiology as described above) contribute to classification of sleep deprivation, and should be included if possible. Our results thus emphasize the importance of using multiple data types to classify causes of HDS, as the combination of all three data types always provided the highest classification accuracy. Furthermore, our results also imply that studies that use only a single cause of HDS (or do not differentiate between different causes of HDS) are not optimal, as different causes of HDS evoke different physiological and behavioral reactions in drivers.

### Classification Approaches

We tested two classification approaches: classifying all four causes of HDS independently (see section “Independent Classification of Each Cause of HDS”) and classifying each cause of HDS with full information about whether the other three causes of HDS are present. We expected that the latter approach would be more accurate, as it would allow classifiers to better account for the effects of other causes of HDS. However, this was not the case – the results of the two approaches were essentially the same, as evidenced by **Tables 3**, **5**. This implies that multi-stage classifiers (which, e.g., first identify environmental conditions and use this as a basis for inference of driver cognitive/affective states) would not be more effective than parallel single-stage classifiers. Still, we acknowledge that our approach (presence/absence of other causes of HDS used as binary inputs to classifier) was somewhat rudimentary, and that more advanced multi-stage classification schemes may achieve better results. For example, a three-stage classifier could first divide drivers into “good” and “bad” ones based on driver characteristics, then infer the environmental conditions (e.g., traffic density, weather) based on vehicle kinematics, and finally infer the driver’s internal state based on physiology.

### Next Steps

The ultimate goal of identifying the causes of HDS is to use this as a basis for responding to hazardous states in real vehicles. Our study indicates that it is possible not only to automatically detect the presence of HDS, but also to classify the internal or environmental cause of this hazardous state. Additionally, the use of physiological sensors greatly increases the classification accuracy. While the demanding environments could be detected by other means (for example, high traffic density could be detected via external traffic monitoring networks), physiological measurements provide an estimate of the driver’s actual mental state, providing a better estimate of whether the driver is distracted than would be achieved with only external measurements. Furthermore, while our study used laboratory-grade physiological sensors, we believe that similar accuracies could be achieved with recently developed physiological sensors built into the car, such as HR sensors built into the steering wheel (Jung et al., 2014) or respiration sensors built into the driver seat (Dziuda et al., 2012). Thus, the approaches used in our study could be transferred both to other simulated driving environments and to real cars.

We believe that, as the next step, our algorithms should be combined with intelligent decision-making systems. Since our classification algorithms allow the specific cause of HDS to be identified, an intelligent vehicle could use this information to decide how to react to the drivers’ hazardous states. For example, if the system detects that the driver is using a cell phone, it could provide increasing warnings to the driver (Yan et al., 2015) or even require both hands to be placed on the steering wheel. Conversely, if it detects sleep deprivation, it could instruct the driver to pull over and rest before continuing. To support such intelligent decision-making, we have already modified our classification algorithms to function in real-time, and preliminary tests indicate that a classification decision is available less than a second after the 4-min data collection interval has ended.

However, further research into decision-making algorithms is likely necessary to take full advantage of our strategy to identify the causes of HDS. Most state-of-the-art safety monitoring systems for intelligent cars have relatively simple responses to detected HDS [e.g., warning sound in case of driver inattention (Yan et al., 2015)], and new decision-making methods would have to be developed to carry out different actions for different detected causes of hazardous states. Since the accuracy of classification is never perfect, these new methods will need to decide what feedback to provide to drivers based on uncertain estimates of the cause of a driver’s HDS. In this case, it may be more efficient to utilize probabilistic classifiers (e.g., Bayesian networks) that output not only the cause of the HDS, but also the ‘confidence’ of the classifier. A decision-making method based on artificial intelligence techniques could then balance the potential reward of a correct action (increased driver safety) with potential negative outcomes due to incorrect action (e.g., the driver is further stressed out by an alarm, or the driver turns off the system due to frequent errors). In the end, the decision-making system could even take multiple actions if a HDS is likely due to multiple causes, or could take no action if it is not confident in the HDS classification result.

Once classification methods have been successfully combined with intelligent-decision making algorithms, their ability to reduce the frequency and severity of crashes should be tested over longer simulated driving sessions to determine the methods’ potential benefits before using them in real-world cars. At that point, additional cost-benefit analyses should be done to determine which sensors could be easily removed without decreasing the effectiveness of the system. Once testing in simulated driving environments has been completed, the developed methods could finally be used to increase safety in real-world driving, potentially reducing the high number of deaths and injuries that are caused by distracted driving.

### Study Limitations

Three limitations of the study should be acknowledged. First, we did not use some measurements that are common in analyzing driver states, such as eye tracking, EEG and body gesture monitoring, all of which have the potential to provide significant insight into HDS. While we believe that the current study nonetheless provides very useful insights into potential benefits of combining driving behavior and physiological measurements, future studies should also include other measurements in order to determine whether, for example, the HR and respiration measures used in our study could be omitted in favor of eye tracking.

Second, we performed classification over 4-min intervals in order to allow comparison to other studies on physiology-based detection of cognitive and affective states, which tend to use intervals of 2–5 min (Healey and Picard, 2005; Novak et al., 2012). However, it is possible that this interval is either too short or too long. In real-world settings, even a second of HDS (e.g., distraction) can lead to catastrophic results, suggesting that shorter intervals should be used. On the other hand, some causes of HDS (bad weather, high traffic density) may only induce stress and workload in the user if experienced for a longer period of time, and 4-min intervals may thus be too brief to induce measurable physiological changes in the user. In future studies, we will investigate the effect of different time periods on the accuracy of identifying the cause of the HDS.

Finally, some of the scenarios experienced by participants were relatively uncontrolled and led to differences in participant behavior that were not always quantifiable. For example, in the cell phone scenarios, some participants used their phone to simply read the news while others used it to actively type and send messages. This may have led to different physiological responses between participants, but further analysis is not possible since participants’ specific cell phone behavior was not captured. Future studies could consider using more controlled tasks (e.g., requiring all participants to write the same message) or capturing participants’ behavior in more detail to allow better analysis. At the same time, it should be acknowledged that real-world driver monitoring systems will need to deal with uncontrolled situations and incomplete information about the user’s behavior, and that controlled laboratory tasks may not provide realistic results.

## Conclusion

Our study used three different data sources (driver characteristics, vehicle kinematics, and physiological measurements) to classify the presence or absence of different causes of HDS. The combination of all three data sources was the most accurate, and was able to classify alert vs. drowsy driving with an accuracy of 98.8%, the use of a cell phone with 82.3%, driving in dense vs. light traffic with 91.4%, and driving in clear vs. snowy weather with 71.5%. These accuracies were achieved in an experiment protocol where other causes of HDS may be present or absent, which represents a greater challenge for classification. Thus, our first conclusion is that combining multiple data sources allows us to not only identify the presence of HDS, but also to identify the specific cause(s) of HDS. This could be used by, e.g., intelligent in-car systems to determine how to intervene in order to lead the driver out of the hazardous state.

Not all data sources were equally effective for different causes of HDS: traffic density and weather were most effectively classified with vehicle kinematics while driver characteristics and physiology were effective for drowsiness and cellphone use. Thus, our second conclusion is that neither vehicle kinematics nor physiology alone can provide robust detection of HDS, and that studies that involve only a single cause of HDS are not necessarily representative of real-world situations.

We also attempted to classify the presence or absence of specific causes of HDS based on binary information about what other causes of HDS are present. However, adding this information did not increase the accuracy of classification. Thus, our third conclusion is that multi-stage classifiers (e.g., first identify the driving environment using vehicle kinematics, then identify the driver’s internal state using physiology) are not necessarily more effective than simple single-stage classifiers. However, this should be examined in more detail, as our methods were admittedly somewhat rudimentary.

As the next step, our algorithms for identification of the cause of HDS should be combined with intelligent decision-making systems that could tailor their response to the specific cause of negative driver. Such a combined HDS cause identification and intervention system could then be tested in simulated driving to determine the degree to which it reduces the frequency and severity of accidents.

## Data Availability Statement

The raw data supporting the conclusions of this manuscript are included as a **Supplementary File**. The raw data file contains the different features (vehicle kinematics, physiology, and driver characteristics) for all participants, sessions, and scenarios within each session. To protect participant anonymity, potentially identifiable information (age, gender, height, dominant hand, use of glasses, etc.) have been omitted.

## Author Contributions

AD led the data collection, data analysis, and wrote the majority of the manuscript. SG and MA assisted with the study design, data collection, and data analysis. DN supervised the entire study, led the study design, and contributed to data analysis and manuscript writing. All authors read and approved the final manuscript.

## Disclaimer

The content of this paper is solely the responsibility of the authors and does not necessarily represent the official views of the National Science Foundation or the National Institutes of Health.

## Conflict of Interest Statement

The authors declare that the research was conducted in the absence of any commercial or financial relationships that could be construed as a potential conflict of interest. The reviewer GA and handling Editor declared their shared affiliation.
