# Supplementary material for: Identifying the Causes of Drivers’ Hazardous States Using Driver Characteristics, Vehicle Kinematics, and Physiological Measurements
Source: Front Neurosci. 2018 Aug 14;12:568. doi: 10.3389/fnins.2018.00568 (PMC6102354; doi:10.3389/fnins.2018.00568)
Supplement: Supplementary file 1 [file Data_Sheet_1.docx]

Supplementary Material

Identifying the causes of drivers’ negative states using driver characteristics, vehicle kinematics and physiological measurements

Ali Darzi, Sherif M. Gaweesh, Mohamed M. Ahmed, Domen Novak

*** Correspondence:** Domen Novak: dnovak1@uwyo.edu

# Examples of representative classifiers

All classifiers are validated using the leave-one-out method. Though the presented classification accuracy in the paper is the mean accuracy across all *k* subsets, it is not rational to present the mean classifier coefficients across all subsets. Therefore, classifier coefficients or rules (in case of decision tree (DT) are presented for one representative subset.

## Cell phone vs. no cell phone classification.

Among all three classifier types, logistic regression (LR) shows the highest accuracy for cell phone vs. no cellphone classification. For the representative data subset, the training and testing accuracies in cross-validation are 98.2% and 91.5%, respectively. Table S.1 shows the selected features and the obtained logistic regression coefficients.

**Table S.1.** Best selected features for cell phone use classification using logistic regression. Abs = absolute value, ECG = electrocardiogram, GSR = galvanic skin response, Std = standard deviation.

| **Feature** | **Coefficient** |
| --- | --- |
| Abs (gradient (ECG)) | 2.6926 |
| Mean (respiration rate) | 2.0508 |
| Mean (lane offset) | 2.6869 |
| Difference of tonic GSR | -0.8758 |
| Number of GSR | 1.1414 |
| Mean amplitude of GSR | 0.7175 |
| Mean (HR) | 1.7986 |
| Std (respiration rate) | -0.7553 |
| Std (Throttle) | -1.5769 |
| Std (vertical velocity) | 1.2017 |
| Fluctuation of (slip of front tires) | -1.9901 |
| Constant | -3.1835 |

## Alert vs. drowsy classification

Among all three classifier types, the decision tree (DT) shows the highest accuracy for alert vs. drowsy classification. Different members of the DT family are used: simple, medium, complex and ensemble boosted. Due to the size of the medium and complex DTs, they cannot be presented here. Instead, a simple representative decision tree is shown in Fig. S.1. Furthermore, Table S.2 lists the features selected for classification of all subsets using ensemble boosted DT. For the representative data subset, the training and testing accuracies in cross-validation are 84.2% and 72.0%, respectively.


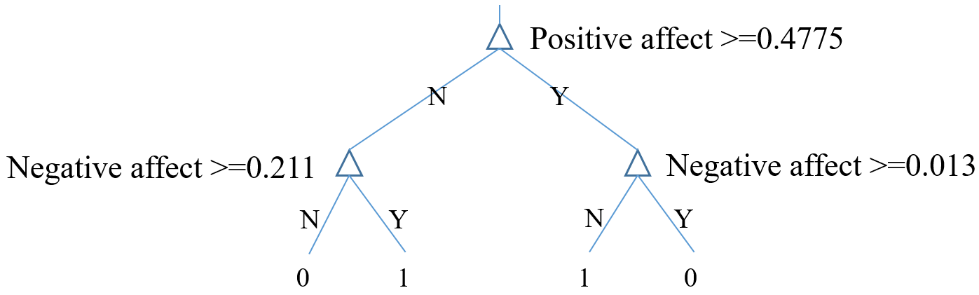


**Figure S.1**. A simple decision tree for alert vs. drowsy classification.

**Table S.2.** Best selected features for alert vs. drowsy classification using Decision Tree

| **#** | **Feature** | **#** | **Feature** |
| --- | --- | --- | --- |
| 1 | Positive Affect | 4 | Stress level |
| 2 | Negative Affect | 5 | Intellect/Imagination |
| 3 | Difference of GSR tonic | 6 | Participant’s Age |

## Low vs. high traffic density classification.

Among all three classifier types, logistic regression (LR) shows the highest accuracy for low vs. high traffic density classification. For the representative data subset, the training and testing accuracies in cross-validation are 99.3% and 100%, respectively. Table S.3 shows the selected features and the obtained logistic regression coefficients.

**Table S.3.** Best selected features for cell phone use classification using logistic regression. Abs = absolute value, ECG = electrocardiogram, RR = respiration rate, HR = heart rate, GSR = galvanic skin response, Std = standard deviation.

| **Feature** | **Coefficient** |
| --- | --- |
| Std (lane number) | 2.8220 |
| Power of low frequencies (HR) | -5.5572 |
| Std amplitude of GSR | 2.3701 |
| Std (respiration rate) | 1.4894 |
| Mean (HR) | 1.9481 |
| Std of inter heart beat interval | 1.2852 |
| Power of high frequencies (HR) | 0.8574 |
| Abs (gradient (ECG)) | 1.9799 |
| Mean (slip of front tires) | -1.7403 |
| Fluctuation of (slip of front tires) | -1.3552 |
| Fluctuation of (slip of back tires) | 1.8332 |
| Constant | -1.2931 |

## Sunny vs. snowy weather classification

Among all three classifier types, the Support Vector Machine (SVM) with linear kernel shows the highest accuracy for sunny vs. snowy weather classification. For the representative data subset, the training and testing accuracies in cross-validation are 93.1% and 87.5%, respectively. Table S.4 shows the selected features and the obtained logistic regression coefficients.

**Table S.4.** Best selected features for classification of alert vs. drowsy classification using support vector machines. HR = heart rate, GSR = galvanic skin response, Std = standard deviation.

| **Feature** |
| --- |
| Mean of tonic GSR |
| Power of low/high (HR) |
| Std (throttle) |
| Std (slip of back tires) |
| Fluctuation of (slip of back tires) |

# Raw data

The raw data supporting the conclusions of this manuscript are included as a Microsoft Excel (.xls) spreadsheet. The file contains the different features (vehicle kinematics, physiology and driver characteristics) for all participants, sessions and scenarios within each session. To protect participant anonymity, potentially identifiable information (age, gender, height, dominant hand, use of glasses etc.) have been omitted.
